# Knowledge About COVID-19 in Brazil: Cross-Sectional Web-Based Study

**DOI:** 10.2196/24756

**Published:** 2021-01-21

**Authors:** Vinícius Henrique Almeida Guimarães, Maísa de Oliveira-Leandro, Carolina Cassiano, Anna Laura Piantino Marques, Clara Motta, Ana Letícia Freitas-Silva, Marlos Aureliano Dias de Sousa, Luciano Alves Matias Silveira, Thiago César Pardi, Fernanda Castro Gazotto, Marcos Vinícius Silva, Virmondes Rodrigues Jr, Wellington Francisco Rodrigues, Carlo Jose Freire Oliveira

**Affiliations:** 1 Institute of Health Sciences Federal University of Triangulo Mineiro Uberaba Brazil; 2 Laboratory of Immunology and Bioinformatics Institute of Biological and Natural Sciences Federal University of Triangulo Mineiro Uberaba Brazil; 3 Institute of Language Studies University of Campinas Campinas Brazil

**Keywords:** COVID-19, coronavirus, perception, knowledge, Brazil, cross-sectional, online survey, health information

## Abstract

**Background:**

COVID-19 is a highly transmissible illness caused by SARS-CoV-2. The disease has affected more than 200 countries, and the measures that have been implemented to combat its spread, as there is still no vaccine or definitive medication, have been based on supportive interventions and drug repositioning. Brazil, the largest country in South America, has had more than 140,000 recorded deaths and is one of the most affected countries. Despite the extensive quantity of scientifically recognized information, there are still conflicting discussions on how best to face the disease and the virus, especially with regard to social distancing, preventive methods, and the use of medications.

**Objective:**

The main purpose of this study is to evaluate the Brazilian population’s basic knowledge about COVID-19 to demonstrate how Brazilians are managing to identify scientifically proven information.

**Methods:**

A cross-sectional study design was used. An original online questionnaire survey was administered from June 16 to August 21, 2020, across all five different geopolitical regions of the country (ie, the North, Northeast, Center-West, Southeast, and South). The questionnaire was comprised of questions about basic aspects of COVID-19, such as the related symptoms, conduct that should be followed when suspected of infection, risk groups, prevention, transmission, and social distancing. The wrong questionnaire response alternatives were taken from the fake news combat website of the Brazilian Ministry of Health. Participants (aged ≥18 years) were recruited through social networking platforms, including Facebook, WhatsApp, and Twitter. The mean distributions, frequencies, and similarities or dissimilarities between the responses for the different variables of the study were evaluated. The significance level for all statistical tests was less than .05.

**Results:**

A total of 4180 valid responses representative of all the states and regions of Brazil were recorded. Most respondents had good knowledge about COVID-19, getting an average of 86.59% of the total score with regard to the basic aspects of the disease. The region, education level, age, sex, and social condition had a significant association (*P*<.001) with knowledge about the disease, which meant that women, the young, those with higher education levels, nonrecipients of social assistance, and more economically and socially developed regions had more correct answers.

**Conclusions:**

Overall, Brazilians with social media access have a good level of basic knowledge about COVID-19 but with differences depending on the analyzed subgroup. Due to the limitation of the platform used in carrying out the study, care should be taken when generalizing the study findings to populations with less education or who are not used to accessing social networking platforms.

## Introduction

COVID-19 is a highly transmissible multi-organ viral disease caused by SARS-CoV-2, a new coronavirus [1]. The most severe cases can be fatal and are present in risk groups that include males, older adults, people who are obese, and patients with other comorbidities [2]. The disease is currently the largest public health issue worldwide, having reached, since March 11, 2020, the status of a global pandemic [3]. The virus can be transmitted from person to person through droplets, aerosols, airborne routes, and contaminated surfaces. The most common symptoms of infection are fever, dry cough, fatigue, headache, loss of smell, and shortness of breath [1]. The disease does not yet have a vaccine or definitive treatment. For this reason, measures such as social distancing, proper hygiene, and the use of individual and collective protective equipment have been instituted by different health authorities, which have been shown to be central to preventing the transmission of the virus and controlling the spread of the disease [4,5]. In addition, knowledge about the infection and its signs and symptoms, whether by the general population or by health professionals, has also been shown to be effective in aiding early diagnosis, better monitoring, and more effective treatment [4].

Brazil is a country of continental dimensions and, in addition to its geographical and cultural differences found within its borders, presents significant economic and educational vulnerabilities. Since the appearance of the country’s first case of COVID-19, much discussion has ensued on how best to face the disease, especially with regard to social distancing (eg, whether “vertical isolation” or “horizontal isolation” should be practiced), the use of medications without World Health Organization approval (eg, azithromycin, ivermectin, and hydroxychloroquine), and the monitoring of the disease from the onset of symptoms to the admission of the patient to a hospital specialized in treating the infection [6]. In addition to these discussions and related challenges, miraculous “cures,” inconsistency between policies and scientific evidence, conspiracy theories, and increases in fake news have been widely disseminated on social networks, which has caused confusion among the general population and hindered the fight against the disease.

Many countries have sought to understand all there is to know about the pandemic to better fight this dangerous disease. For this reason, several researchers have conducted studies to track the public’s knowledge and misperceptions regarding COVID-19. Studies with this focus have already been conducted among general and specific populations in mainland China [7], Colombia [8], Hong Kong [9], India [10], Iran [11], Israel [12], the United Kingdom [13], and the United States [14,15], among others. These studies, despite the differences in their findings, clearly demonstrate that populations present a certain level of knowledge about COVID-19. On the other hand, these studies have also revealed how much the disease has had economic, psychosocial, and behavioral impacts that also need to be mitigated. At the time of writing (September 2020), Brazil is the third country with the highest number of confirmed cases and has had more than 140,000 deaths from the disease [16]; however, no studies have evaluated the population’s basic knowledge about COVID-19. Thus, this study seeks to evaluate the public’s knowledge and misperceptions about COVID-19 and the preventive measures adopted to date in the country.

## Methods

### Participants

A cross-sectional anonymous online survey (see Multimedia Appendix 1) was carried out using Google Forms, a service for form and questionnaire creation that is free for everyone who has a Google account. This tool allows for the creation of different types of questions, the collection and organization of the responses received, and generation of spreadsheets and graphs of the final data in real time.

In an attempt to make possible the implementation of the research and aiming at easy access to the online survey, respondents were recruited via the divulgation of information regarding the research on the university’s and researcher’s social medias. With the expectation of reaching the largest possible number and diversity of people, the disclosure was made in four of the main social medias used in Brazil: WhatsApp, Instagram, Facebook, and Twitter. For a better representation of the overall Brazilian population, the researchers also used promotional tools on these social networks—paying for advertisements to enable the survey form to reach different audiences from all regions of the country. According to the Brazilian Institute of Geography and Statistics, the population of Brazil in 2020 reached 211.8 million, of whom around 134 million have access to the internet. Thus, according to statistical analysis, a sample number of 2500 participants would be representative of the population using internet in the country, with a 2% margin of error and 95% confidence level. Still, in addition to the stages of confusion and risk of bias control, the data was evaluated to 64% above the estimated sample number, making a total of 4100 participants distributed in the 5 macroregions of Brazil.

The online form was available for about 2 months between June 16 and August 21, 2020, and can be found in its full version in Multimedia Appendix 1. This specific time period was selected because it was the peak of the pandemic’s “first wave” in the country (ie, the period when, for the first time, the pandemic reached a peak in cases and deaths) [17]. The 2-month availability was due to the geographical extent of Brazil and the need for representativity of the population from each region. As a country of continental dimensions, the disease has not spread homogeneously throughout the country. In addition, cities far from the research centers that the researchers belonged were more difficult to access.

This study was approved by the ethics and research committee of the Federal University of Triângulo Mineiro in Minas Gerais State, Brazil. Upon access to the web-based survey form, respondents were provided with an explanation as to the purpose of the research as well as the prerequisites for participation. Potential participants could decide freely whether to participate in the study. Those aged ≥18 years, the target study participants, were then asked to select the option of electronically signing the free and informed consent form.

In Brazil, the legislation defines 18 years as the age of majority, making the individual fully capable to respond by himself. Considering that the research was online and there was an urgent need for it to be carried out at the height of the COVID-19 spread in the country, there would be a greater bureaucratic obstacle if the research had to include younger individuals because more documents would need to be filled out and analyzed by the ethics committee. This group is also considered vulnerable, meaning that it would be necessary to have authorization from a guardian older than 18 years. Therefore, we chose to recruit only participants aged ≥18 years. If the participants consented, they were directed to answer the questions on the form. There was no financial compensation for participants who responded to the survey; thus, participation was voluntary and anonymous.

The survey was elaborated on considering data from the official website of the Brazilian Ministry of Health, the country’s highest health authority responsible for organizing and preparing public plans and policies aimed at health across the country. Thus, the basis for outlining the questions and the correct alternatives was developed according to the information available on the COVID-19 webpage of the Brazilian Ministry of Health [18]. To elaborate on the incorrect alternatives and seeking to address some difficulties faced by Brazilians in terms of understanding the disease, the website of the Brazilian Ministry of Health was used. This website compiles some fake news about the disease to discuss its flaws from a scientific point of view and to clarify it for the population [19].

As the objective was to understand the Brazilian residents’ knowledge about COVID-19, the questionnaire that participants had access to was in the official language spoken and written in all regions of the country, the Portuguese language. After the completion of data collection, an English translation of the questions and alternatives was provided for this publication (see Multimedia Appendix 1).

### Data Collection and Quality Assurance

At the end of the data disclosure period, we stopped collecting questionnaire responses on Google Forms. We then obtained a spreadsheet with all the survey data, with each row representing the responses of a participant and each column representing the answers to a question. Using a filter tool available in Excel (Microsoft Corporation), it was possible to identify and exclude both the responses of participants who declared they were younger than 18 years and the responses of participants who declared they were not residents of Brazil.

**Figure 1:**
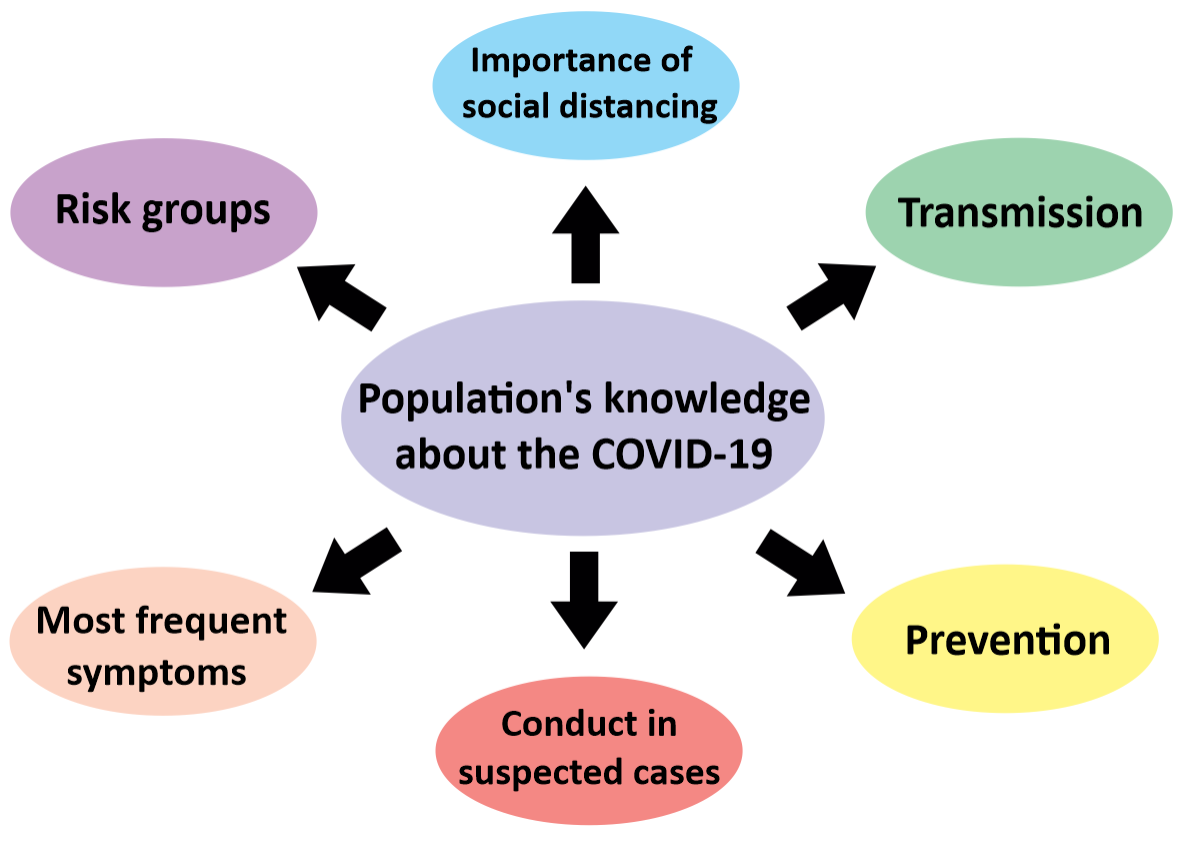
Basic health knowledge investigated in the study.

In the survey form, participants were asked 26 questions that were divided into five main blocks of themes: (1) general information about COVID-19 (ie, questions about transmission, most common symptoms, conduct in case of infection, risk groups, and social distancing), (2) sharing information about the disease (ie, questions about how participants obtain information about COVID-19, what information they receive and share, and how they check and analyze the news they find on social media), (3) identification of misinformation (analyzing whether the participants recognize fake news about the disease), (4) economic and social impact of the pandemic (ie, questions about the main family fears and challenges, and what most hinders the search for information about the disease), and (5) sociodemographic information about the participants (ie, age, sex, education level, region of residence, profession, whether they were recipients of government benefits, and number of people living in the same household). In this study, only the questions that directly assessed the participants’ basic knowledge in health education about COVID-19 (see Figure 1) were referred to in the analysis; specifically, this included questions on the forms of transmission (question 1), main symptoms of the disease (question 2), conduct in a suspected case (question 3), identification of risk groups (question 4), understanding of social distancing (question 5), and disease prevention (question 12). The other questions did not directly evaluate knowledge about health and disease, and were not referred to in the analysis. The number of alternatives for each question took into account the proportion of fake news available on the website of the Ministry of Health. Thus, questions that addressed more fake news verified by the website were considered, proportionally, as issues of greater confusion among the population, receiving more alternatives and presenting greater participation in the final score. Before the evaluation of the participants’ responses, a pilot test with 200 participants (randomized by automation) was carried out to ensure reliability. In our study, the Cronbach alpha was .66 for general information about COVID-19, .94 for sharing information about the disease, .88 for identification of misinformation, .70 for economic and social impact, and .68 for sociodemographic information about the participants. Quality assurance was accomplished by checking, by at least two independent evaluators, the data collection, extraction and entry to the software, and data analysis.

To count the final score of each participant in the six questions chosen for analysis, we classified the questions based on their format (checkboxes or multiple choice). Questions 1, 2, 4, and 12 were arranged in a checkbox format, and the participant could select more than one alternative. Questions 3 and 5 were in the multiple choice format, with the participant being able to choose only one alternative.

In the questions arranged in checkboxes, all alternatives, correct or incorrect, were evaluated. There were four possibilities for selecting alternatives: (1) the alternative was correct and was selected, (2) the alternative was correct and was not selected, (3) The alternative was incorrect and was not selected, and (4) the alternative was incorrect and was selected. In options 1 and 3, the participant got the choices right and scored 1 point. In options 2 and 4, the participant made a mistake in the choices and scored 0 points. That is, the participants score 1 point both when choosing which alternative is correct and when choosing which is incorrect. Thus, the maximum score for these questions corresponded to the total number of alternatives in the question and indicated that the participant marked the correct alternatives and did not mark the incorrect ones, scoring 1 point for all alternatives. Question 1 was composed of 7 alternatives, with a maximum value of 7 points; question 2 had 9 alternatives, with a maximum value of 9 points; question 4 had 10 alternatives, with a maximum value of 10 points; and question 12 had 11 alternatives, with a maximum value of 11 points.

In multiple choice questions, there is a limitation in the number of checked alternatives and only one could be selected. There were only two possibilities for selecting alternatives: (1) the alternative was correct and was selected and (2) the alternative was incorrect and was selected. In option 1, the participant got it right and scored 1 point. In option 2, the participant made a mistake and scored 0 points. Therefore, the participants only scored points when they selected the correct alternative. Thus, the maximum score for these questions was always 1 and indicated that the participant chose the correct alternative. Therefore, although questions 3 and 5 have 3 alternatives each, the maximum value of these questions was 1, indicating the selection of the correct alternative.

Taking into account the maximum value that questions in checkboxes and multiple choice could receive, the participant could score between 0 and 39 points in total. Simply put, Higher scores had more correct answers, meaning more knowledge about COVID-19 was evident. Scores between 0-9 were regarded as *poor knowledge* about the disease, scores between 10-19 were regarded as *regular knowledge*, scores between 20-29 were regarded as *good knowledge*, and scores between 30-39 were regarded as *optimal knowledge*. The required knowledge investigated in this study can be considered basic, as they mainly concerned practical aspects of people’s day-to-day life, not entering into the theoretical or scientific merits of a complex multi-organ disease such as COVID-19.

At the end of the questionnaire, participants had access to some links that directed them to sources of scientifically safe information about the disease, such as the official website of the Brazilian Ministry of Health [18,19] and the website of a main research institution in infectious diseases in the country, the Oswaldo Cruz Foundation (FIOCRUZ) [20]. Participants also had the possibility to check their own answers, the right and wrong answers, and the explanation for each alternative.

### Statistical Analysis

The data were tabulated using Excel and analyzed using SPSS 21 (IBM Corp) and GraphPad Prism 7.0 (GraphPad Software, Inc). The data were evaluated for their distribution (using D’Agostino-Pearson and Shapiro-Wilk normality tests), and the variances were compared (using the *F* test and Bartlett test). Unpaired tests to compare the distributions of the different variables were used (Kruskal-Wallis with Dunn multiple comparisons test and Mann-Whitney *U* test). The hypotheses were tested using chi-square, Fisher exact, and chi-square with Yates correction tests. To assess the association measures, odds ratios (Baptista-Pike) with their respective confidence intervals were used in the definitive analysis.

To assess the effect of associations between the tested variables in the third table (ie, transmission, symptoms, conduct in suspected infection, risk groups, social distancing, and prevention), the lowest scores (the poor outcomes) were compared with the other scores (the best outcomes) between the descriptions for each variable. For the grouped variables, the scores were normalized in relative frequencies and were compared with the scores up to 50%, with the others (above 50%) between the descriptions for each variable. Multivariate analysis was performed to determine the hierarchical groupings of the different variables. After adjusting the proximity matrix using the squared Euclidean distance, the results were plotted on a dendrogram. Spearman test was used to investigate correlations. The significance levels in all statistical tests were less than .05 (5%) [21].

## Results

**Figure 2:**
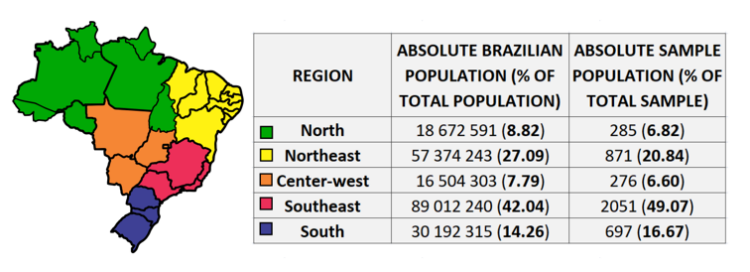
Distribution of the Brazilian population by region and its relationship with the distribution of the study population.

A total of 4436 responses were received; however, 17 were excluded from the analysis due to the respondents having been from other countries, and 239 were excluded for having been filled out by people younger than 18 years, thus leaving a total of 4180 valid responses. Of these valid responses, 2051 (49.07%) came from the Southeast, 871 (20.84%) from the Northeast, 697 (16.67%) from the South, 285 (6.82%) from the North, and 276 (6.60%) from the Central-West geopolitical regions of Brazil (Figure 2). The average age of respondents was 34.57 years; 2937 (70.26%) were women, 2040 (48.80%) held a bachelor’s degree or above, and 3504 (83.83%) lived with a maximum of four people in the same house. Among the respondents, 3252 (77.80%) stated that they did not receive any kind of government assistance. Most (n=3641, 87.11%) had not traveled to other countries in the past year. These and other demographic information are shown in Table 1.

**Table 1:** Demographic characteristics of participants.

Characteristic		Participants (N=4180), n (%)
**Sex**		
	Male	1243 (29.74)
	Female	2937 (70.26)
**Age (years)**		
	18-19	315 (7.54)
	20-29	1718 (41.10)
	30-39	761 (18.21)
	40-49	583 (13.95)
	50-59	558 (13.35)
	≥60	245 (5.86)
**Region**		
	North	285 (6.82)
	Northeast	871 (20.84)
	Central-West	276 (6.60)
	Southeast	2051 (49.07)
	South	697 (16.67)
**Education**		
	Middle and high school	2140 (51.20)
	Higher and postgraduate education	2040 (48.80)
**Household size**		
	1 person	354 (8.47)
	2 people	841 (20.12)
	3 people	1177 (28.16)
	4 people	1132 (27.08)
	5 people	442 (10.57)
	6 people or more	234 (5.60)
**Receives government social assistance**		
	Yes	928 (22.20)
	No	3252 (77.80)

Figure 2 illustrates the distribution of the participants by region. For the total score, measured between 0 and 39 possible points, the average score of the participants was 33.77 points, varying between 20 and 39 points in total, depending on the respondent. This means that, on average, the participants reached 86.59% of the total possible score. Table 2 shows the distribution of responses (true or false) for each response alternative to the questions presented. In the table, for each alternative, we can observe if the item was considered false or true (in parenthesis) and the number of people who appropriately marked it.

**Table 2:** Questionnaire of knowledge about COVID-19.

Questions		Participants (N=4180), n (%)
**What are the main forms of transmission of COVID-19?**		
	Through sneezing, coughing, or talking to infected people (true)	4082 (97.66)
	Direct contact with domestic animals (false)	132 (3.16)
	Bringing hand to face after touching contaminated surfaces (true)	3765 (90.07)
	Bites from contaminated insects (false)	26 (0.62)
	Taking filtered water in cities with many cases of infection (false)	232 (5.55)
	Using products that came from China, where the coronavirus appeared (false)	85 (2.03)
	Contact with contaminated people (eg, kiss, hug, or handshake; true)	3954 (94.59)
**What are the three most common symptoms of COVID-19?**		
	Diarrhea and vomiting (false)	418 (10.00)
	Skin wounds (false)	19 (0.45)
	Persistent fatigue (true)	1250 (29.90)
	Stuffy nose (false)	270 (6.46)
	Fever (true)	3775 (90.31)
	Shortness of breath (false)	3410 (81.58)
	Cough (true)	3192 (76.36)
	Headache (false)	1144 (27.37)
	Sneezing (false)	542 (12.97)
**What is the possible conduct after infection?**		
	The virus is not that dangerous, so you can continue your life normally (false)	6 (0.14)
	You should be isolated at home and seek help if you feel short of breath or get worse (true)	3338 (79.86)
	You must immediately go to the hospital to seek medical attention (false)	836 (20.00)
**Which risk groups are most likely to get infected?**		
	People with heart or kidney problems (true)	2806 (67.13)
	People with vision problems (eg, blindness or myopia; false)	19 (0.45)
	Wheelchair users (false)	85 (2.03)
	People with respiratory diseases and smokers (true)	4070 (97.37)
	Older adults (true)	3995 (95.57)
	People with cancer (true)	2668 (63.83)
	Adolescents and young adults (false)	59 (1.41)
	People with diabetes or high blood pressure (true)	3778 (90.38)
	Pregnant women (true)	1531 (36.63)
	There are no risk groups (false)	24 (0.57)
**Importance of social distancing**		
	Necessary (true)	4101 (98.11)
	Makes no difference (false)	66 (1.58)
	Harmful (false)	13 (0.31)
**Which alternatives are true about COVID-19?**		
	There is already a vaccine against COVID-19 (false)	226 (5.41)
	Wearing gloves and masks for everyday activities decreases the chance of becoming infected with the virus (true)	3590 (85.89)
	Gargling with warm water, salt, and vinegar prevents coronavirus (false)	72 (1.72)
	Hot water or tea kills the coronavirus (false)	46 (1.10)
	70% gel alcohol kills the coronavirus (true)	3678 (87.99)
	Chloroquine protects people from becoming infected with the coronavirus (false)	114 (2.73)
	There are already drugs that cure COVID-19 (false)	143 (3.42)
	Soap, sanitary water, liquid alcohol, and common detergents kill the coronavirus (true)	3404 (81.44)
	Drinking alcohol kills the virus (false)	8 (0.19)
	Social distancing has no scientific proof (false)	140 (3.35)
	Once the person has had the coronavirus infection, they cannot have it again because they are immune (false)	567 (13.56)

**Figure 3:**
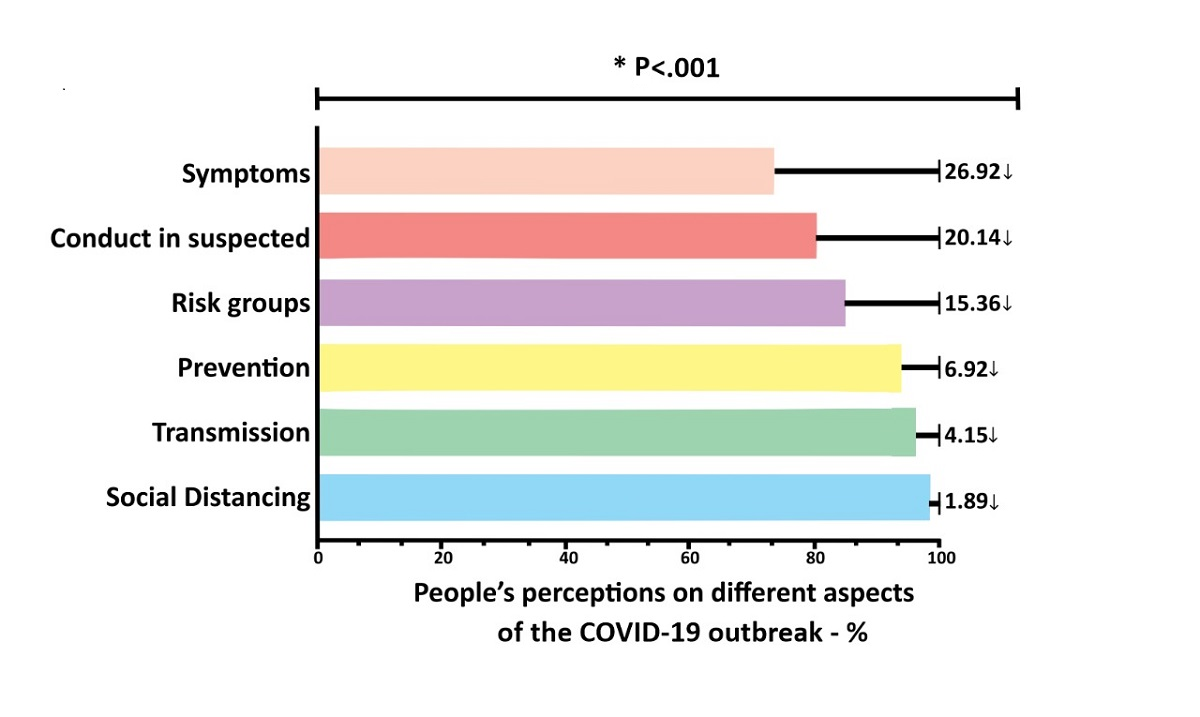
Distribution and association among COVID-19 health education indicators. The differences or similarities between the participants’ levels of correct answers on questions regarding the symptoms of COVID-19, the conduct of those suspected of SARS-CoV-2 infection, risk groups, disease prevention, disease transmission, and perception of social distancing. The relative distribution, in percentages, of the levels of right and wrong answers for each variable and comparisons between them is demonstrated. *Statistically significant differences between groups (Kruskal-Wallis test with Dunn multiple comparison tests).

**Figure 4:**
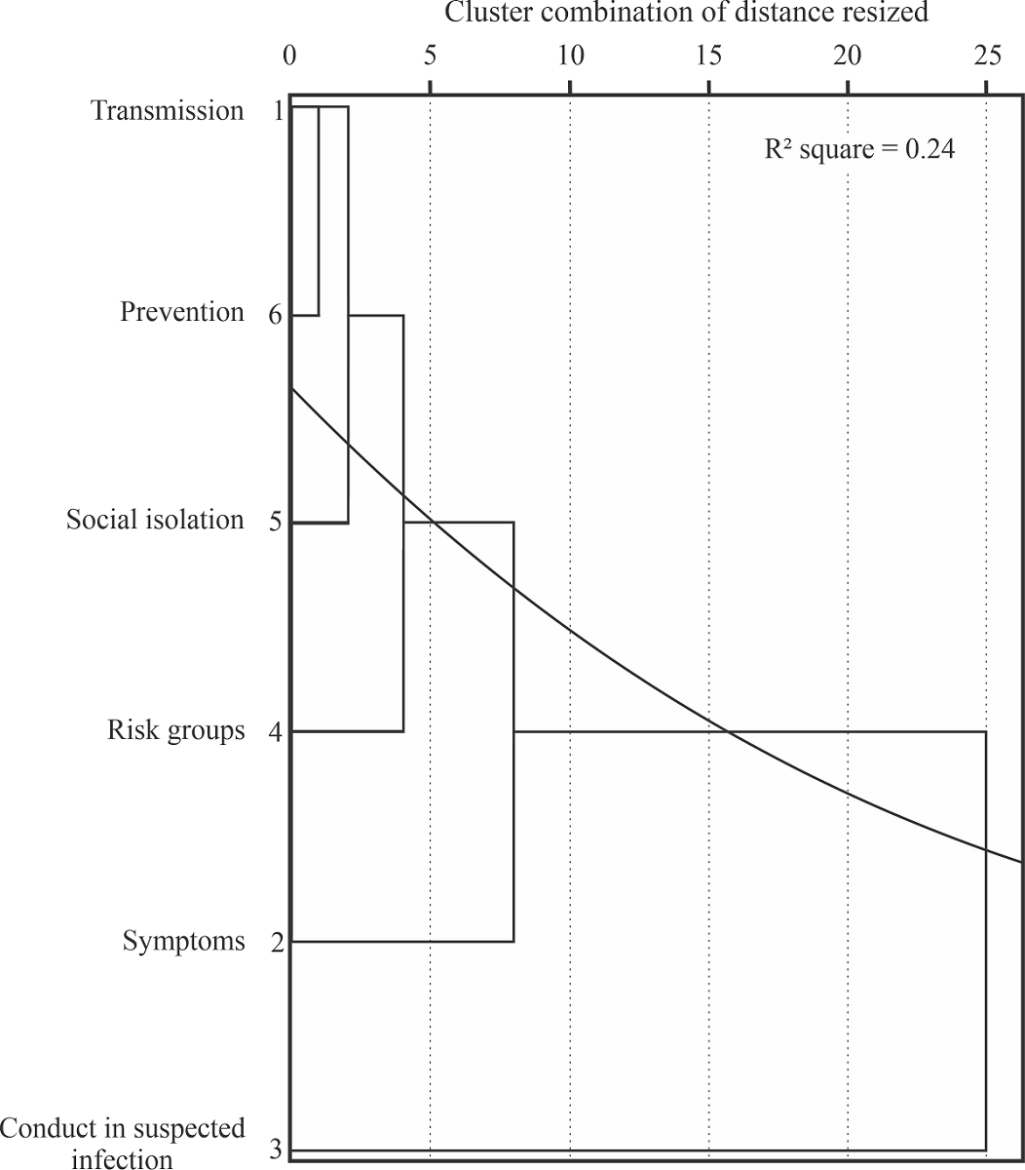
Research variables were combined in clusters and, after obtaining the square Euclidean distance and plotting on a dendrogram, the keys indicated the similarity between the variables evaluated in the survey. The central line indicates an adjustment by the square R2.

After determining the sociodemographic profile of the participants (Table 1) and the survey questions (Table 2), the percentage of the population’s knowledge about COVID-19 with regard to the different research variables (Figure 1) was evaluated, including transmission, symptoms, conduct for suspected infection, risk groups, perception of social distancing, and prevention. Overall, the participants had a good perception of the COVID-19 outbreak since the percentage of correct answers was above 90% and never below 70% for some of the variables evaluated. There was no participant that had a poor or regular knowledge score. On the other hand, of the 4180 responses, 252 (6.03%) had good knowledge scores and 3928 (93.97%) had optimal knowledge scores. When the level of perception between each variable was assessed, a statistically significant difference (*P*<.001) was found between them, with knowledge about symptoms being the parameter with the lowest understanding by the respondents (73.08% of correct responses). This limited understanding of the symptoms of the disease was 14 times lower than the knowledge about the importance of social distancing. There was an important lack of understanding concerning the conduct to be taken in cases of suspected SARS-CoV-2 infection (20.14%), risk groups (15.36%), disease prevention (6.92%), and disease transmission (4.15%; Figure 3). It is important to note that all the variables studied were linked in the range of the squared Euclidean distance, and an intimate relationship was observed between social distancing, transmission, and prevention. On the other hand, in general, choosing the right or wrong answer did not respect these relationships, as the similarities of the average connections between the groups were not consistent (quadratic *R*^2^=0.22; Figure 4).

The step immediately after evaluating all the participants together was to investigate their perception of COVID-19 by considering their sociodemographic profiles and each research variable. Thus, each of the respondent’s health education indicators was evaluated considering, for example, sex (male and female), education level (middle/high school and higher/postgraduate education), age (18-83 years), socioeconomic vulnerability (receiving or not receiving government support), and the number of people per residence (1-6). These indicators were evaluated for their respective distributions for each profile (unpaired), and the odds ratios between the worst outcomes (lowest score) were compared with those between the best outcomes. We also grouped the media scores of all parameters (ie, transmission, symptoms, conduct in the case of an infection, risk groups, and social distancing) and evaluated the odds ratios of them in each population profile (ie, sex, education level, age, socioeconomic vulnerability, people by residence, and Human Development Index [HDI]) to have a percentage of correct answers of up to 50%, as described in the Methods section and Table 3.

**Table 3:** Sample statistical analysis based in sociodemographic profiles.

Variables			Mean (SD)	M-W^a^ test	K-W^b^ test	Spearman *r* (95% CI)	OR^c^ (95% CI)	*P* value
**Transmission**								
	**Sex**			1,765,703	N/A^d^	N/A	0 (0 to 2.73)	*.01* ^e^
		Male	6.68 (0.63)					
		Female	6.72 (0.64)					
	**Education level**			2,041,706	N/A	N/A	1.91 (0.22 to 27.66)	*<.001*
		Middle and high school	6.66 (0.69)					
		Higher/postgraduate	6.76 (0.572)					
	**Age (years)**			N/A	68.82		N/A	*<.001*
		18-19	6.71 (0.76)^f,g^			N/A		N/A
		20-29	6.80 (0.50)^g^			N/A		N/A
		30-39	6.66 (0.69)^f,h^			N/A		N/A
		40-49	6.60 (0.71)^h^			N/A		N/A
		50-59	6.63 (0.70)^f,h^			N/A		N/A
		≥60	6.60 (0.74)^f,h^			N/A		N/A
		18-83	34.57 (14.01)			–0.08 (–0.12 to 0.05)		*<.001*
	**Socioeconomic vulnerability**			1,412,808	N/A	N/A	1.75 (0.12 to 15.09)	*<.001*
		RGS^i^	6.62 (0.75)					
		Not RGS	6.73 (0.60)					
	**People in residence (n)**			N/A	N/A	–0.02 (–0.05 to 0.01)	N/A	.26
		1-40	3.35 (1.61)					
	**HDI** ^j^			N/A	N/A	0.10 (0.07 to 0.13)	N/A	*<.001*
		0.683-0.850	0.779 (0.04)					
**Symptoms**								
	**Sex**			1,825,002	N/A	N/A	1.03 (0.50 to 2.10)	.99
		Male	6.57 (1.08)					
		Female	6.58 (1.06)					
	**Education level**			2,171,656	N/A	N/A	1.92 (0.95 to 3.99)	.73
		Middle and high school	6.57 (1.09)					
		Higher/Postgraduate	6.58 (1.04)					
	**Age (years)**			N/A	18.66		N/A	*.002*
		18-19	6.52 (1.15)^f,h^			N/A		N/A
		20 to 29	6.65 (1.02)^f^			N/A		N/A
		30 to 39	6.54 (1.03)^f,h^			N/A		N/A
		40 to 49	6.57 (1.13)^f,h^			N/A		N/A
		50 to 59	6.51 (1.12)^h^			N/A		N/A
		≥60	6.44 (1.08)^h^			N/A		N/A
		18-83	34.57 (14.01)			–0.10 (–0.12 to 0.05)		*<.001*
	**Socioeconomic vulnerability**			1,398,824	N/A	N/A	2.61 (1.30 to 5.16)	*<.001*
		RGS	6.45 (1.11)					
		Not RGS	6.61 (1.05)					
	**People in residence (n)**			N/A	N/A	–0.01 (–0.04 to 0.02)	N/A	.66
		1-40	3.35 (1.61)					
	**HDI**			N/A	N/A	0.07 (0.04 to 0.10)	N/A	*<.001*
		0.683-0.850	0.779 (0.04)					
**Conduct in suspected infection**								
	**Sex**			1,809,913	N/A	N/A	0.95 (0.80 to 1.12)	.53
		Male	0.80 (0.40)					
		Female	0.80 (0.40)					
	**Education level**			2,149,210	N/A	N/A	0.91 (0.78 to 1.06)	.21
		Middle and high school	0.81 (0.39)					
		Higher/postgraduate	0.79 (0.41)					
	**Age (years)**			N/A	295.40		N/A	*<.001*
		18-19	0.87 (0.33)^f^			N/A		N/A
		20-29	0.90 (0.30)^f^			N/A		N/A
		30-39	0.78 (0.42)^g^			N/A		N/A
		40-49	0.70 (0.46)^h^			N/A		N/A
		50-59	0.68 (0.47)^h^			N/A		N/A
		≥60	0.55 (0.50)^k^			N/A		N/A
		18-83	34.57 (14.01)			–0.25 (–0.28 to –0.22)		*<.001*
	**Socioeconomic vulnerability**			1,473,256	N/A	N/A	1.15 (0.97 to 1.38)	.11
		RGS	0.78 (0.41)					
		Not RGS	0.80 (0.40)					
	**People in residence (n)**			N/A	N/A	0.01 (–0.02 to 0.04)	N/A	.73
		1-40	3.35 (1.61)					
	**HDI**			N/A	N/A	0.12 (0.09 to 0.15)	N/A	*<.001*
		0.683-0.850	0.779 (0.04)					
**Risk groups**								
	**Sex**			1,641,327	N/A	N/A	2.37 (0.89 to 6.32)	*<.001*
		Male	8.31 (1.29)					
		Female	8.53 (1.27)					
	**Education level**			2,010,364	N/A	N/A	0.71 (0.25 to 2.06)	*<.001*
		Middle and high school	8.37 (1.31)					
		Higher/postgraduate	8.56 (1.24)					
	**Age (years)**			N/A	33.51		N/A	*<.001*
		18-19	8.14 (1.38)^f,g^			N/A		N/A
		20-29	8.52 (1.20)^f,g,h^			N/A		N/A
		30-39	8.56 (1.29)^f,h^			N/A		N/A
		40-49	8.52 (1.29)^f,g,h^			N/A		N/A
		50-59	8.37 (1.38)^g,h^			N/A		N/A
		≥60	8.29 (1.39)^g^			N/A		N/A
		18-83	34.57 (14.01)			0.02 (–0.01 to 0.05)		.13
	**Socioeconomic vulnerability**			1,449,382	N/A	N/A	0.58 (0.13 to 2.26)	.06
		RGS	8.39 (1.31)					
		Not RGS	8.49 (1.27)					
	**People in residence (n)**			N/A	N/A	–0.02 (–0.06 to 0.01)	N/A	.10
		1-40	3.35 (1.61)					
	**HDI**			N/A	N/A	0.01 (–0.02 to 0.04)	N/A	.50
		0.683-0.850	0.779 (0.04)					
**Social distancing**								
	**Sex**			1,780,394	N/A	N/A	3.21 (2.06 to 5.00)	*<.001*
		Male	0.96 (0.19)					
		Female	0.99 (0.11)					
	**Education level**			2,181,870	N/A	N/A	0.98 (0.63 to 1.53)	.92
		Middle and high school	0.98 (0.13)					
		Higher/postgraduate	0.98 (0.14)					
	**Age (years)**			N/A	18.93		N/A	*.002*
		18-19	0.97 (0.16)^f,h^			N/A		N/A
		20-29	0.99 (0.11)^f^			N/A		N/A
		30-39	0.97 (0.16)^f,h^			N/A		N/A
		40-49	0.98 (0.14)^f,h^			N/A		N/A
		50-59	0.99 (0.12)^f^			N/A		N/A
		≥60	0.95 (0.22)^h^			N/A		N/A
		18-83	34.57 (14.01)			–0.03 (–0.06 to 0.00)		*.03*
	**Socioeconomic vulnerability**			1,507,802	N/A	N/A	0.96 (0.55 to 1.63)	.88
		RGS	0.98 (0.13)					
		Not RGS	0.98 (0.14)					
	**People in residence (n)**			N/A	N/A	–0.02 (–0.05 to 0.01)	N/A	.16
		1-40	3.35 (1.61)					
	**HDI**			N/A	N/A	–0.01 (–0.04 to 0.02)	N/A	.42
		0.683-0.850	0.779 (0.04)					
**Prevention**								
	**Sex**			1,803,511	N/A	N/A	7.1 (1.06 to 92.31)	.50
		Male	10.21 (1.03)					
		Female	10.25 (0.94)					
	**Education level**			2,010,310	N/A	N/A	0.95 (0.15 to 6.09)	*<.001*
		Middle and high school	10.16 (1.01)					
		Higher/postgraduate	10.32 (0.91)					
	**Age (years)**			N/A	37.61		N/A	*<.001*
		18-19	10.05 (1.01)			N/A		N/A
		20-29	10.35 (0.85)			N/A		N/A
		30-39	10.17 (1.03)			N/A		N/A
		40-49	10.25 (0.90)			N/A		N/A
		50-59	10.15 (1.06)			N/A		N/A
		≥60	10.07 (1.29)			N/A		N/A
		18-83	34.57 (14.01)			–0.03 (–0.06 to 0.00)		.07
	**Socioeconomic vulnerability**			1,407,623	N/A	N/A	0.00 (0 to 3.51)	*<.001*
		RGS	10.17 (0.95)					
		Not RGS	10.26 (0.97)					
	**People in residence (n)**			N/A	N/A	0.01 (–0.02 to 0.04)	N/A	.54
		1-40	3.35 (1.61)					
	**HDI**			N/A	N/A	0.10 (0.03 to 0.09)	N/A	*<.001*
		0.683-0.850	0.779 (0.04)					
**Grouped variables**								
	**Sex**			1,680,078	N/A	N/A	1.25 (1.01 to 1.56)	*<.001*
		Male	33.55 (2.45)					
		Female	33.86 (2.42)					
	**Education level**			1,981,133	N/A	N/A	1.69 (1.37 to 2.08)	*<.001*
		Middle and high school	33.56 (2.55)					
		Higher/postgraduate	33.99 (2.28)					
	**Age (years)**			N/A	110.10		N/A	*<.001*
		18-19	33.27 (2.41)^f,k^			N/A		N/A
		20-29	34.21 (2.11)^h^			N/A		N/A
		30-39	33.69 (2.57)^g^			N/A		N/A
		40-49	33.63 (2.57)^f,g^			N/A		N/A
		50-59	33.32 (2.58)^f,k^			N/A		N/A
		≥60	32.92 (2.84)^k^			N/A		N/A
		18-83	34.57 (14.01)			–0.10 (–0.12 to –0.05)		*<.001*
	**Socioeconomic vulnerability**			1,334,021	N/A	N/A	1.50 (1.19 to 1.87)	*<.001*
		RGS	33.39 (2.53)					
		Not RGS	33.88 (2.39)					
	**People in residence (n)**			N/A	N/A	–0.02 (–0.05 to 0.01)	N/A	.26
		1-40	3.35 (1.61)					
	**HDI**			N/A	N/A	0.10 (0.07 to 0.13)	N/A	*<.001*
		0.683-0.850	0.779 (0.04)					

^a^M-W: Mann-Whitney.

^b^K-W: Kruskal-Wallis.

^c^OR: odds ratio.

^d^N/A: not applicable.

^e^Italics indicate statistically significant difference.

^f^Statistically significant difference between these groups.

^g^Statistically significant difference between these groups.

^h^Statistically significant difference between these groups.

^i^RGS: receiving government support.

^j^HDI: Human Development Index.

^k^Statistically significant difference between these groups.

With regard to sex, women had a better understanding and greater knowledge about transmission (0.60% more; *P*=.01) and risk groups (2.65% more; *P*<.001), with no difference in odds ratios. Regarding the understanding of the importance of social distancing, in addition to a better average of female performance (3.13% more; *P*<.001), there was also a higher probability of low performance for men (OR 3.21, 95% CI 2.06-5.00). A similar result was observed when the average score of all parameters was grouped, with a better average accuracy by women (*P*<.001) and a greater probability of incorrect responses by men (OR 1.69, 95% CI 1.01-1.56; Table 3).

When the population’s perception about COVID-19 was assessed taking into account the education level, no significant differences were found between the groups concerning knowledge about symptoms, actions in case of suspected SARS-CoV-2 infection, and social distancing (all *P*>.05). On the other hand, the higher education and postgraduate group obtained a better average for knowledge about transmission (1.50% more; *P*<.001), risk groups (2.27% more; *P*<.001), and prevention (1.57% more; *P*<.001). When the average scores of all parameters were grouped, the higher/postgraduate group had a better average performance (*P*<.001), and the middle and high school group had a higher probability of lower performance (OR 1.69, 95% CI 1.37-2.08; Table 3).

The age of the participants influenced the understanding and comprehension of all COVID-19 health education indicators (all *P*<.05). The younger participants had a better understanding. In addition, a negative and significant correlation (all *P*<.05) was observed in relation to knowledge about transmission, symptoms, conduct in suspected infection, risk groups, and social distancing. On the other hand, concerning the indicator of prevention, no significant correlation was observed (all *P*>.05). After grouping the average scores for all parameters, the negative and significant correlation between age and percentage of correct answers was maintained (*P*<.001), with the greatest average difference observed in the group 60 years and older, and in the group aged 20-29 years (percentage of correct answers 3.92% higher; *P*<.001; Table 3).

The influence of socioeconomic vulnerability on the population’s perception of COVID-19 was estimated through the investigation of participants who received or did not receive any type of financial support or resource from the government during the pandemic. Regardless of whether or not they received any financial support, no statistically significant differences were found with regard to the understanding of conduct in case of suspected SARS-CoV-2 infection (*P*=.11), risk groups (*P*=.06), and the importance of social distancing (*P*=.88). On the other hand, a higher percentage of correct answers was observed in the population with better socioeconomic conditions for knowledge about transmission (percentage of correct answers 1.66% higher; *P*<.001), symptoms (percentage of correct answers 2.48% higher; *P*<.001), prevention (percentage of correct answers 0.88% higher; *P*<.001), or even when the average scores of all parameters were grouped (percentage of correct answers 1.47% higher; *P*<.001). In addition, a greater probability for a lower percentage of correct answers was observed among people with socioeconomic vulnerability regarding symptoms (OR 2.61, 95% CI 1.30-5.16) and when the average scores of all parameters were grouped (OR 1.50, 95% CI 1.19-1.87; Table 3).

The relationship between the number of people per household and correct answers was also assessed; however, no statistically significant association was found for any of the examined questions (all *P*>.05). Correlations were also investigated regarding the HDI and the percentage of correct answers. Positive and significant correlations (all *P*<.001) were found in all evaluated variables, except for knowledge about risk groups (*P*=.50; Table 3).

Taking into account the vast territorial extent of Brazil and its cultural, climatic, and political influences, among others, we also evaluated the possible differences in correct answers of questions related to health education on COVID-19 by region (Table 4). Regardless of region, a significant percentage of the respondents answered questions incorrectly about COVID-19. In the specific cases of knowledge about the main risk groups and social distancing, the percentage of incorrect responses was similar in all regions (average incorrect responses 15.62% and 2.40%, respectively). Knowledge of the population in each region, on the other hand, about transmission, symptoms, conduct in cases of suspected infection, prevention, and the average score of all variables together showed significant differences between regions (Table 4).

**Table 4:** Sample statistical analysis based in geopolitical regions.

Variables		North	Northeast	Central-West	Southeast	South	*P* value (K-W^a^ test)
**Transmission**							*<.001* ^b^
	Mean (SD)	6.57 (0.74)^c^	6.62 (0.71)^d^	6.68 (0.72)^e^	6.79 (0.53)^f^	6.64 (0.70)^g^	
	CV^h^ (%)	11.34	10.72	10.77	7.88	10.52	
	Reduction (%)	–6.14	–5.43	–4.57	–3.00	–5.14	
**Symptoms**							*<.001*
	Mean (SD)	6.38 (1.12)^c^	6.53 (1.06)^c,e^	6.45 (1.10)^c,e^	6.64 (1.03)^d,e^	6.60 (1.13)^e^	
	CV (%)	17.57	16.24	17.00	15.55	17.13	
	Reduction (%)	–29.11	–27.44	–28.33	–26.22	–26.67	
**Conduct in cases of suspected infection**							*<.001*
	Mean (SD)	0.67 (0.47)^c^	0.74 (0.44)^d^	0.72 (0.45)^c,d^	0.86 (0.39)^e^	0.77 (0.42)^d^	
	CV (%)	70.84	58.70	61.76	40.51	54.40	
	Reduction (%)	–33.00	–26.00	–28.00	–14.00	–23.00	
**Risk groups**							.52
	Mean (SD)	8.32 (1.40)	8.42 (1.35)	8.50 (1.20)	8.50 (1.21)	8.45 (1.36)	
	CV (%)	16.79	16.06	14.11	14.23	16.15	
	Reduction (%)	–16.80	–15.80	–15.00	–15.00	–15.50	
**Social distancing**							.28
	Mean (SD)	0.98 (0.14)	0.98 (0.13)	0.96 (0.19)	0.98 (0.13)	0.98 (0.13)	
	CV (%)	14.69	13.25	19.42	13.18	13.80	
	Reduction (%)	–2.00	–2.00	–4.00	–2.00	–2.00	
**Prevention**							*<.001*
	Mean (SD)	10.03 (1.12)^c^	10.19 (0.98)^c^	10.06 (1.08)^c^	10.33 (0.89)^d^	10.19 (1.02)^c^	
	CV (%)	11.19	9.60	10.72	8.65	9.99	
	Reduction (%)	–8.82	–7.36	–8.55	–6.09	–7.36	
**Grouped variables**							*<.001*
	Mean (SD)	32.94 (2.78)^c^	33.48 (2.56)^d,e^	33.37 (2.42)^c,e^	34.10 (2.20)^f^	33.64 (2.62)^d^	
	CV (%)	8.44	7.64	7.27	6.44	7.79	
	Reduction (%)	–15.54	–14.15	–14.44	–12.56	–13.74	

^a^K-W: Kruskal-Wallis.

^b^Italics indicate statistically significant difference.

^c^Statistically significant difference between these groups.

^d^Statistically significant difference between these groups.

^e^Statistically significant difference between these groups.

^f^Statistically significant difference between these groups.

^g^Statistically significant difference between these groups.

^h^CV: coefficient of variation.

Respondents from the North region of Brazil had the highest percentage of wrong answers on transmission (6.14%), followed by those from the Northeast (5.43%), South (5.14%), Central-West (4.57%), and Southeast (3%). Respondents from the North region also presented a higher percentage of wrong answers on questions about the symptoms (29.11%), conduct to be taken in case of suspected infection (33%), prevention (8.82%), and when all the variables were grouped (15.54%). The Northeast was the second region in the number of wrong answers about COVID-19, with percentage of incorrect responses of 27.44%, 26%, 7.36%, and 14.15% for symptoms, conduct in cases of suspected infection, prevention, and when all variables were grouped, respectively. The Central-West was third in percentage of wrong answers with 28.33%, 28%, 8.55%, and 14.44% for symptoms, conduct in case of suspected infection, prevention, and when all variables were grouped, respectively. Meanwhile, the South had percentage of incorrect responses of 26.67%, 23%, 7.36%, and 13.74% for symptoms, conduct in case of suspected infection, prevention, and grouping of all variables, respectively. On the other hand, the Southeast had the lowest percentage incorrect responses, with percentages of 26.22%, 14.00%, 6.09%, and 12.56% for symptoms, conduct in case of suspected infection, prevention, and grouping the average score of all parameters, respectively (Table 4).

## Discussion

### Sample Data

We assessed the Brazilian population’s basic knowledge about COVID-19. To this end, an online survey was made available that allowed people 18 years or older and who use social media for communication and information to test their knowledge about the disease and infection. The findings were elucidating, as this method of gathering information allowed for the evaluation of people from different regions of Brazil, different social groups, of different ages, and with different education levels (ranging from people with only basic education to people with graduate degrees). The survey respondents were predominantly female, young (younger than 40 years), from the Southeast region, and composed of people who did not receive government assistance. Women’s greater concern with health [22] and the massive use of social media by young individuals with better social and economic conditions are aspects that may explain the predominant final configuration of the findings [23]. In general, the respondents presented satisfactory basic knowledge about COVID-19, scoring an average of 86.59% of the maximum possible survey score but with statistically significant differences depending on the question, group, or region analyzed. That is, there were differences in the groups analyzed that showed that knowledge about the disease, although reasonable, differed depending on the respondent. Similar studies in other countries have been carried out and have presented participants with characteristics close to those obtained in our study, that is, satisfactory basic knowledge of the disease but with inequalities depending on the analyzed group [7,11,15,24]. This was the case in a study carried out in China [7], in which participants scored an average of 90% of the total possible score with a predominantly young female sample and with just over half of the interviewees having completed undergraduate and graduate courses.

With respect to social media disseminating metrics, a total of R $803.76 (US $155.77) were invested so that 5908 clicks on the form link could be reached. This means, on average, that R $0.14 (US $0.03) per click was spent. A total of 239,414 people were reached, generating 349,320 impressions. That is, 2.47% of the people who were reached with the dissemination accessed the link. These metrics show that despite a relatively low investment per click, searches for paid ads like this arouse the interest of a minority of the people reached. Considering that the research producers also collaborated on disseminating the form on social media, we observed that it was filled out by 4180 participants, meaning that not all of those who clicked on the link followed the form until its completion. This low adherence was expected due to, among other reasons, the fact that the metrics are proportional to the perception of the institution’s credibility [25]. Thus, as science in Brazil still has a lack of credibility among the population, especially due to problems of communication between science and the practical appropriation of the scientific knowledge, it was already expected that there would be a minority of responses in relation to the total number of individuals impacted by the dissemination [26].

### Main Findings

As illustrated in Figure 3, there was a statistical difference among the number of correct answers according to the investigated question. More correct answers were recorded on questions related to the importance of social distancing, treatment, and prevention. We observed that, similarly, these same items were answered correctly in the United States, China, Pakistan, the United Kingdom, and India [6,7,11,24], showing that these are issues with global levels of comprehension. The lowest scores occurred on questions concerned with knowledge about the symptoms of the disease, risk groups, and conduct for patients suspected of infection. Participants overestimated shortness of breath, did not recognize some risk groups such as pregnant women, and demonstrated more misconceptions regarding the conduct in suspected cases. This difference, depending on the questioned item, was also found by other studies in other countries. However, the wrong questions vary; in Brazil, as described, the question with the fewest correct answers was that related to symptoms, while in a survey carried out in Pakistan [24], the question related to transmission received the fewest correct answers. This indicates that these different scores may depend on the specific aspect about COVID-19 being investigated, and these aspects may vary according to the country studied.

In our research, as shown in Table 3, the three symptoms that the respondents most believed to be connected with COVID-19 were fever, dry cough, and shortness of breath. The first two symptoms are correct; however, the third is not: shortness of breath has occurred in a minority of COVID-19 cases, and the correct response alternative would be the symptom of fatigue [27]. Although symptoms such as diarrhea, skin wounds, vomiting, stuffy nose, shortness of breath, headache, and loss of taste or smell may be present, they are less frequent [27]. Because the question was limited to the three most common symptoms (persistent tiredness, fever, and cough), the alternatives that encompassed these less common symptoms were false. It is important to note that when checking the answers participants were informed that, although these other symptoms were not the most frequent, they could show up. Research conducted in India, the United Kingdom, and the United States [10,13] showed a similar situation (ie, the replacement of fatigue by shortness of breath as one of the three main symptoms of the disease). The media dissemination of more serious cases and of deaths related to shortness of breath, hospitalization, and the use of pulmonary ventilators may have led the general population to believe that dyspnea is a common manifestation of the disease. It is important to note that this perception can negatively influence people’s behavioral conduct and create a dualistic view of COVID-19 (ie, it can lead individuals to believe that they are infected only when shortness of breath is present; otherwise, they are healthy, which is not entirely true). In other words, this perception can be worrying in relation to milder and asymptomatic cases, which normally do not exhibit shortness of breath. In the absence of the manifestation of this symptom, these groups may not conduct themselves appropriately because they believe they are not infected, thereby becoming potential transmitters of the disease.

The second question that received the most incorrect answers was related to a patient’s conduct in case of suspected SARS-COV-2 infection. In Brazil, the Ministry of Health has relayed that in case of suspected COVID-19, individuals need to stay at home in isolation and only seek health services in certain situations such as when symptoms are more severe. This course of action is in line with the recommendations of the World Health Organization. However, at the beginning of the pandemic, the increased concern of the population caused hospitals to be filled with people with mild symptoms who sought medical care even though their chances of complications were low, for they neither were from risk groups nor had worrying symptoms. This overload ended up bringing crowds to health services, including people without COVID-19 who were concerned with any sign common to the disease, contributing to the spread of the virus in the population. Surveys conducted in China and India [7,10], places where the same course of action has also been endorsed by their governments, have had participants show high levels of correctness in answers. This means that, regarding one’s conduct in case of suspected COVID-19 infection, efforts to disseminate the relevant correct information have resulted in a relatively adequate awareness among the population; however, in Brazil, it seems that it is necessary to reinforce this awareness.

The third question that received the most incorrect answers was related to identifying risk groups among the general population who were more likely to deal with the most severe forms of the disease. Some risk groups were adequately recognized by the 4180 participants; however, other risk groups were less recognized, as was the case for people with heart or kidney problems (n=2806, 67.13%), people with cancer (n=2668, 63.83%), and pregnant women (n=1531, 36.63%). At the beginning of the pandemic, there was still no certainty about the inclusion of pregnant women in the risk group. Months later, the Brazilian Ministry of Health officially included this part of the population in this group. It is possible that this initial confusion may have influenced the correct recognition of risk groups. This factor may have a negative impact by decreasing the precaution around these groups, which require more attention. In China and Pakistan [7,24], older adults, people who are obese, and patients with chronic diseases have also been recognized by several studies as being the most common risk groups. In the United States and the United Kingdom [13], older adults were also recognized as a risk group, followed by adults with health problems. However, 53.8% of Americans and 39.1% of British people also recognized children as a risk group, which, from a scientific point of view, is incorrect [28]. People therefore more easily recognize important risk groups such as older adults and people with chronic diseases. However, others are being forgotten, such as pregnant women in Brazil, or are being incorrectly assigned, such as children in the United States and the United Kingdom. With regard to the questions that were answered correctly the most in our research (ie, those regarding prevention, transmission, and social distancing), we observed similar items in surveys conducted in China, India, Pakistan, the United Kingdom, and the United States, and the findings of these surveys showed that their participants’ comprehension levels had already reached that of many nations in the world [7,10,13,24].

Regarding sex, women obtained more correct answers than men in the questions on general knowledge about COVID-19—a finding that conflicts with the literature: in the United States and the United Kingdom [14], no difference in the correctness of survey answers was observed between men and women. In Pakistan [24], men were more correct than women; in China [7], as well as in Brazil, women were more correct than men. The lack of similarity in findings among countries and the differences between the sexes seem to indicate that our findings with respect to sex may be underdetermined by other characteristics including economic, social, and cultural development. We also separated the study participants as undergraduates, graduates, and those who were neither. In this sense, participants who had higher educational qualifications were observed to be better informed about the disease and infection. In China and Pakistan [7,24], similar findings were found, indicating that a higher level of education indicates a higher level of knowledge about COVID-19. These findings underscore that investments in education (in addition to contributing to scientific development) create a more informed population, as is the case with regard to COVID-19 and, moreover, any disease that may affect the general population, whether as a pandemic or not.

Differences were identified when the participants were grouped by age, with the younger participants tending to have more correct answers than those who were older, especially when comparing older adults with individuals aged 20-29 years. In Pakistan, this correlation was also found [24], but in China [7], this was not the case; older adults were the second most successful group. Although our data do not correspond worldwide, it is possible that in some countries younger individuals, who tend to have had earlier experience with the internet and social networks, have a more critical perception with the information conveyed on social networks.

Concerning the aspects of socioeconomic vulnerability, HDI, and regional differences, no research was found in the literature that has investigated these aspects. Thus, we consider our study findings to be unique and important in more accurately understanding people’s knowledge of the pandemic. The information we have obtained evidences the reasoning that more socioeconomically advanced groups have greater knowledge about COVID-19. That is, a correlation was found between the lack of government assistance and the highest HDI in the region with the largest number of correct answers regarding COVID-19. In practical terms, development and income may be predictors for better levels of access to and interpretation of information, including those related to COVID-19, thus enabling a better understanding of the disease.

The different regions of the country had different levels of success, which reflects the important social and economic differences between the regions [29]. This highlights the need to develop specific public policies for each location, with greater emphasis on conduct awareness in case of suspected infection in the North region and identification of the main symptoms in other regions of the country.

### Practical Applications

It is important to consider that one of the key points that began this research was the significant spread of fake news, conspiracy theories, and contradictory orientations among the population. Fake news is not a phenomenon exclusive to the COVID-19 pandemic; it has been verified in other contexts, especially in political elections [30]. However, in the pandemic, their impact can be dramatic; when spread in a sustained manner, they have a disruptive effect on the preventive measures necessary to combat COVID-19. With less prevention, more people become contaminated, greater overload occurs in the health system, and consequently, more deaths are accounted. This is especially problematic in Brazil, a transitional country whose public health system has a burden of diseases and lack of resources [31].

In Brazil, studies have indicated that 9 out of 10 people have read or heard at least one source of false information about COVID-19, while 7 out of 10 believe in at least one uninformative source about the disease [32]. This significant proportion of misinformation is not a mere disinterested product without scientific knowledge. Fake news has several purposes in validating points of view that are incompatible with science but serve political, economic, and even criminal interests. As fake news spreads six times faster than true information, producers can create this content to generate network traffic for financial return with advertising, or there may even be scams asking for money for respected scientific institutions to fight COVID-19 [32,33].

However, when analyzing the study population, it was possible to verify that there is a satisfactory knowledge about COVID-19 when true information and fake news are mixed. Participants demonstrated that they were able to differentiate the two types of information. Thus, although more studies are needed, it is possible to suggest that the impact of fake news on the knowledge of COVID-19 in the population of our study was limited. This does not mean that fake news has a limited impact on the Brazilian population in general, as this study did not fully analyze it nor did it select all the fake news that exists among the population; only a few of the main ones selected by the Brazilian Ministry of Health were used, and their verification had already been made public in advance. This result shows a certain effectiveness in campaigns against the Brazilian government’s lack of information at the beginning of the pandemic and underscores the importance of continuing this action. In spite of this, it is necessary to consider that the knowledge assessed was considered basic, that is, excessively technical aspects of a complex disease such as COVID-19 were not addressed. The alternatives were based on practical aspects disclosed by the Ministry of Health that the population could use in their daily life.

Nevertheless, even with the good theoretical knowledge demonstrated by the population of this study, the practice is still not represented in the population’s behavior. In Brazil, the practice of social distancing is unsatisfactory; agglomeration cases are recurrent; and, although efficient, preventive measures still do not show significant adherence by the population [34]. Thus, there is a gap between theorical knowledge and satisfactory practice. In a way, this shows that the problem of a lack of adherence to preventive measures cannot be attributed exclusively to fake news. In other words, the lack of knowledge is not the only factor that impacts the generation of an effective practice against the pandemic in Brazilians who use social networks. To consolidate the practice of fighting COVID-19, in addition to producing knowledge, it is necessary to provide more conditions for its practical implementation. The need to investigate and to correct other social, political, economic, and cultural conditions that are preventing a disciplined coping with the pandemic is evident, not exclusively attributing the responsibility for low public engagement to the fake news.

In addition, our findings are useful to political authorities, journalistic or media groups, and even to social media. This is because the findings unfolded here diagnose some weak spots in the population’s knowledge about COVID-19. Despite how satisfactory the general knowledge of the disease may be, failures were observed in certain groups such as men, older adults, and undereducated people; in locations such as those with the lowest HDI; and in aspects of the disease, such as the most common symptoms, conduct in suspected cases, and identification of risk groups. Some of these findings have even been confirmed in studies from other countries, showing a similarity that goes beyond continents. Ultimately, public and private institutions responsible for informing the population need to focus their efforts on these shortcomings.

We also demonstrated that investments in education and socioeconomic improvements can have a positive impact on the knowledge and actions of the population, which can be useful not only in coping with COVID-19 but also in other diseases or possible future pandemics. These two pillars, in addition to allowing investigations that improve the effort to fight and treat the disease, are themselves capable of educating citizens more immune to fake news.

### Limitations

Online surveys have some limitations. Participants, for example, could search for answers on the internet or choose random alternatives to quickly complete the questionnaire that would impair some of the data.

As this study had no deadline and was voluntary and anonymous, participants were free in their decision to engage. They were also warned that their knowledge would not be exposed, leaving them more comfortable to answer the questions, avoiding any related bias. Despite this and taking into account the state of the pandemic and social isolation in Brazil, dissemination through social media through a form by Google Forms proved to be a viable solution for assessing knowledge about COVID-19.

The findings of this study only apply to people who use social platforms, can read and write, present some level of knowledge about the pandemic, and have compatible electronic equipment to answer the survey. In other words, Brazil is a country that still has a high rate of illiteracy [35], and a large portion of the population does not have the internet and equipment necessary to access the online survey. Thus, although the survey findings represent an important portion of the population, it cannot be generalized as being applicable to the entire Brazilian population. In addition, the survey was optional, which may indicate that a large part of the responses came from participants with a greater interest in information concerning the disease. This could have an influence in the participants’ good performance. To reduce this limitation, we sought to evaluate a high number of participants, which eventually brought greater representativeness to the sample.

Finally, for this publication, the questionnaire data were translated from Portuguese (the official language of Brazil) into English. Some translation problems could change certain interpretations of sentences. To avoid that, we submitted the revised version of the manuscript to a professional academic English editing service.

### Conclusions

The Brazilian population with access to social networks demonstrated satisfactory basic knowledge about COVID-19. Despite this, there were differences among the issues, groups analyzed, and regions of the country. In general, participants had better knowledge about prevention, transmission, and social distancing but made more mistakes in identifying the main symptoms, risk groups, and correct conduct in cases of infection. Better performances were also observed among women, young people between 20 and 29 years of age, undergraduates and graduates, and those who did not receive any type of government assistance. In addition, a positive correlation was identified between the best HDI and the level of knowledge about the disease.

## Appendix

Multimedia Appendix 1 The complete version of the form given to participants.

## Abbreviations

HDI: Human Development Index
