# Supplementary material for: Knowledge About COVID-19 in Brazil: Cross-Sectional Web-Based Study
Source: JMIR Public Health Surveill. 2021 Jan 21;7(1):e24756. doi: 10.2196/24756 (PMC7822056; doi:10.2196/24756)
Supplement: Multimedia Appendix 1 [file publichealth_v7i1e24756_app1.docx]

**Supplementary Material**

Test your knowledge about COVID-19!

Hello! This test contains questions that allow you to evaluate your knowledge about COVID-19, questions about how you get informed about the disease and also about the impact the pandemic may have had in your life. At the end of the survey, you will be able to verify the correctness of your answers. The test is fast and anonymous. It is also important to remember that this test is being conducted by the Federal University of Triângulo Mineiro (UFTM). The average time required of you is five minutes as there are only 15 questions.

You are invited to participate in the study “People’s Knowledge and Their Sources of Information About COVID-19.” The overall goal of this study is to understand how people normally get informed about the COVID-19 pandemic. Using this information, we intend to create strategies that would be beneficial in the fight against this virus. As a participant, you will be asked to answer a few questions. In addition, your name will not appear in the study as any information you provide will be kept anonymous. You will also not suffer any kind of financial loss, embarrassment, discomforting procedure, or life risk. Several questions will enquire about how the coronavirus has impacted your life. You can abandon the questionnaire and quit at any time.

INFORMED CONSENT TERM - I have read the clarification above and understand the purpose of the study. I agree to participate.

What do you know about the coronavirus and COVID-19?

Reminder: the coronavirus is a type of virus that causes the COVID-19 disease.

1. - Can you identify how the coronavirus spreads?

Through sneezing, coughing, or talking to an infected person, or direct contact with domestic house animals (like dogs or cats).

Touching contaminated surfaces and touching the face. Contaminated insect bites

Drinking filtered water in cities with many COVID-19 cases

Using products that have come from China, where the coronavirus had arisen

Contact with contaminated people (e.g., through kissing, hugging, or handshaking)

1. - Can you identify COVID-19’s three most common symptoms? Diarrhea and vomiting

Skin wounds Persistent tiredness Stuffy nose

Fever

Shortness of breath Cough

Headache Sneeze

1. - What would you say to someone possibly infected with the coronavirus?

The virus is not so dangerous, so you can continue your life normally.

You should stay at home.

Look for help if you feel breathless or if the symptoms worsen.

You must get hospital care immediately.

4 - Do you know who is at higher risk from coronavirus? People with heart or kidney problems

People with sight problems (e.g., blindness or myopia) Wheelchair users

People with respiratory diseases (e.g., asthma, bronchitis, or emphysema) and seniors who smoke

People with cancer Teenagers and young adults

People with diabetes or high blood pressure Pregnant women

There is no risk group

1. - To fight against COVID-19, social isolation is…

Necessary

Useless

Prejudicial

How do you share information about the coronavirus and COVID-19?

1. - What media do you use to obtain information about the coronavirus?

Television

WhatsApp Radio Facebook Instagram Twitter YouTube

Ministry of Health mobile applications

News portals (UOL, Terra, Globo, etc.) Alternative or local media websites Official government websites Research articles

1. - Do you share information about coronaviruses? Yes

No

1. - The information you receive or share about the coronavirus are usually...

Texts (short or long) Videos

ImagesAudios

1. - How do you share information about the coronavirus? Through WhatsApp

Through social media (e.g., Facebook, Instagram, Twitter) Talking in person

I do not share information about the coronavirus.

1. - What do you usually do when you receive interesting information about the coronavirus?

I pay attention to see if the person or the group who shared the information is trustworthy.

I check the date when that information was released.

I verify the source/author.

I search that information somewhere else.

I promptly share the information because it can be very important for others to know.

11 - What information about the coronavirus do you NOT share? I do not share information without a source/an author.

I do not share information signed by a source/an author that I do not know.

I do not share information from politicians I did not vote for or that I do not trust.

I do not share information shared by traditional media, like important newspapers and TV stations.

I do not share information shared by humor websites/pages.

I do not share information shared by the health ministry.

I do not share information shared by public universities.

I do not share information that contains errors in language, words in CAPITAL LETTERS, or exaggerated use of punctuation (e.g., !!!!!! or ?!?!?!)

I do not share long information that I did not read or watch entirely.

I do not share information that defames individuals or groups of people.

I do not share information received from people with political opinions different from mine.

I do not share any information.

Are you able to identify between true and false information about coronavirus and COVID-19?

Test your knowledge now!

12 - Which of the alternatives below are true?

A coronavirus vaccine already exists.

The use of gloves and masks in day-to-day activities decreases the chance of contamination. Gargling with warm water, salt, and vinegar prevents coronavirus.

Water or hot tea kill coronavirus.

70% gel alcohol kills coronavirus.

Chloroquine protects people from coronavirus contamination. There are already drugs that cure COVID-19.

Soap, bleach, liquid alcohol, and common detergents kill coronavirus. Drinking alcohol kills the virus.

Social isolation has no scientific evidence.

Once a person has been infected with coronavirus, he or she cannot catch it again because he or she becomes immune.

We want to hear from you too!

We want to know what worries you the most with so many uncertainties and difficulties in Brazil.

13 - What are your three biggest fears related to the coronavirus pandemic?

Being contaminated by the virus and getting sick

Friends and family getting sick Water/power/food shortages

The worsening of the economic situation

Lack of public security, such as increases in burglaries and muggings

That proper medicine or a vaccine are never found

Excessive price raising

Having to cancel or delay events such as weddings, parties, trips, etc.

1. - What gets in your way when you search for coronavirus information?

False information (fake news)

Too much information Contradictory statements Authorities’ speeches Celebrities’ speeches

1. - What are the three biggest challenges faced by you and your family?

Little space in the house for too many people

Difficulty buying medicine

Impossibility to go to bars, restaurants, and leisure spaces Difficulty buying food

Not being able to access health professionals

Taking care of the children, who cannot go to school Prejudiced college or school activities

Not being able to exercise

You want to know all the answers, right? You’re almost there! Just fill in the data below and you will be able to check all your results (remember that the data will be kept anonymous).

- How old are you?
- What is your sex?
  - Male
  - Female
- What is your occupation?
- Are you a health graduate or are you taking a course in this area?
  - No
  - Yes, and I have taken courses in microbiology, parasitology, and infectious diseases.
  - Yes, but I have not taken courses in microbiology, parasitology, or infectious diseases.
- What is your educational level?
  - Primary school
  - High school
  - College
  - PhD
- In which city do you live?
- In which state do you live?
- Including you, how many people live in your house?
- Did you travel somewhere outside Brazil in the last year?
  - No
  - Yes, to other countries in South America
  - Yes, to other countries outside South America
- Do you receive any kind of social benefit?
  - No
  - Yes, family aid
  - Yes, emergency aid
  - Other
- How is the pandemic affecting your family’s budget?
  - It is not affecting because the money we receive or have in reserve remains the same.
  - A little because the money we receive or have in reserve decreased, but it did not affect our standard of living
  - Moderately because we still receive or have some reserve money, the pandemic has already affected our standard of living.
  - A lot because the money we receive or have in reserve has already decreased significantly, leaving little or forcing our family to take on debts.
  - Severely, we no longer receive money or have it in reserve, depending on government aid or aid from people in solidarity.
